# The Overexpression of Keratin 23 Promotes Migration of Ovarian Cancer via Epithelial-Mesenchymal Transition

**DOI:** 10.1155/2020/8218735

**Published:** 2020-11-01

**Authors:** Meng Ren, Yan Gao, Qi Chen, Hongyu Zhao, Xiaoting Zhao, Wentao Yue

**Affiliations:** Central Laboratory, Beijing Obstetrics and Gynecology Hospital, Capital Medical University, Beijing 100026, China

## Abstract

**Background:**

Keratin 23 (KRT23) is a new member of the KRT gene family and known to be involved in the development and migration of various types of tumors. However, the role of KRT23 in ovarian cancer (OC) remains unclear. This study is aimed at investigating the function of KRT23 in OC.

**Methods:**

The expression of KRT23 in normal ovarian and OC tissues was determined using the Oncomine database and immunohistochemical staining. Reverse transcription quantitative polymerase chain reaction assay was used to analyze the expression of KRT23 in normal ovarian epithelial cell lines and OC cell lines. Small interfering RNA (siRNA), wound healing assay, and transwell assay were conducted to detect the effects of KRT23 on OC cell migration and invasion. Further mechanistic studies were verified by the Gene Expression Profiling Interactive Analysis platform, Western blotting, and immunofluorescence staining.

**Results:**

KRT23 was highly expressed in OC tissues and cell lines. High KRT23 expression could regulate OC cell migration and invasion, and the reduction of KRT23 by siRNA inhibited the migration and invasion of OC cells *in vitro*. Furthermore, KRT23 mediated epithelial-mesenchymal transition (EMT) by regulating p-Smad2/3 levels in the TGF-*β*/Smad signaling pathway.

**Conclusions:**

These results demonstrate that KRT23 plays an important role in OC migration via EMT by regulating the TGF-*β*/Smad signaling pathway.

## 1. Introduction

Ovarian cancer (OC) is one of the most common malignant diseases that threaten the health of women worldwide [1]. Despite continuous improvements in surgical skills and chemotherapy drugs, the death rate still accounts for 5% of women, and the mortality rate still ranks first among all gynecological tumors [2]. The low survival rate and poor prognosis associated with OC are mostly due to cancer invasion and migration. Therefore, it is imperative to further explore the molecular mechanisms underlying OC progression and migration in order to improve the clinical outcomes of OC patients.

Keratin (KRT) is the main component of intermediate filaments in epithelial cells, and malignant tumor cells originate from these epithelial cells. The keratin protein family consists of more than 50 members, including 28 type I and 26 type II keratins [3]. Previous studies have shown that keratin plays an important role in cell mechanical integrity and barrier function [4]. Keratin also participates in cellular signal transduction regulation, cell proliferation, protein synthesis, and maintenance of the polarized cellular structure [5]. Several recent studies have reported that abnormal expression of epithelial keratin is associated with the invasion and migration of cancer cells [6–8].

The keratin 23 (KRT23) gene is a new, type I member of the KRT gene family. It is located on human chromosome 17q21.2, which is transcribed into a 1.65 kb mRNA, and then translated into a 48.1 kDa KRT23 protein. Previous studies have revealed aberrations in the expression of KRT23 in different types of human diseases. In 2001, Rogers et al. showed that the expression of KRT23 was higher in the skin, tongue, and breast than in the colon, lung, kidney, and eye [9]. A recent study showed that KRT23 levels were tightly associated with the severity of liver disease [10]. Starmann also reported that KRT23 may act as a useful biomarker for steatohepatitis [11]. Kinast et al. observed a hepatitis C virus-dependent increase in KRT23 mRNA levels in chronic hepatitis C patients [12]. In addition, KRT23 was also reported to be involved in the development and migration of various types of human cancers. Zhang et al. first reported high levels of KRT23 expression in mediated tumor cell differentiation and apoptosis in the human pancreatic cancer cell line AsPC-1 [13]. Furthermore, researchers have identified that KRT23 is localized in the Golgi apparatus in the cytoplasm, highly expressed in colon cancer, and that overexpression of KRT23 promoted colon cancer cell proliferation and migration [14, 15]. Wang et al. detected the levels of tumor-associated antigens by the SEREX method and found that the positive rate of KRT23 expression in the plasma of patients with hepatocellular carcinoma was significantly higher than that in patients with chronic liver disease [16]. Januchowski et al. reported that the expression of KRT23 was increased in methotrexate-resistant variants of the W1 OC cell line but decreased in paclitaxel- and topotecan-resistant variants of the W1 OC cell line, indicating the potential role of KRT23 in drug resistance of OC cells [17]. However, the carcinogenic role of KRT23 in OC has not been elucidated, and its potential mechanism in cancer remains unclear. Growing evidence has demonstrated the underlying mechanism of tumorigenesis and migration of KRT23 in different types of cancers. Birkenkamp-Demtroder et al. reported that dysregulation of KRT23 in colon cancer cells inhibited the expression of key molecules that were related to cell cycle and DNA repair, thereby preventing the proliferation of colon cancer cells and increasing their sensitivity to external stimuli, such as radiation [18]. Zhang found that overexpression of KRT23 increased the activity of telomerase reverse transcriptase, which then promoted colon cancer cell migration [15]. In addition, studies conducted by Liffers et al. suggest that upregulated KRT23 in colon cancer cells could interact with 14-3-3*ε* protein, further regulating the cell cycle and cell apoptosis [19]. Taken together, these studies suggest that the underlying mechanism of KRT23 in cancers requires in-depth exploration.

Hence, in this study, the role of KRT23 in OC was investigated for the first time, and it was identified that KRT23 was overexpressed in OC tissues and OC cell lines. In addition, *in vitro* experiments indicated that knockdown of KRT23 inhibited OC cell migration and invasion. Moreover, KRT23 further regulated the migratory and invasive abilities of OC cells via EMT by influencing the levels of p-Smad2 and p-Smad3. Overall, we demonstrated the role of KRT23 in regulating migration and EMT of OC cells through the TGF-*β*/Smad signaling pathway.

## 2. Materials and Methods

### 2.1. Database Analysis

The expression levels of KRT23 in 329 OC tissues and 19 normal tissues were obtained from the Oncomine database (http://www.oncomine.org). To explore the biological roles of KRT23 in OC, Gene Set Enrichment Analysis (GSEA) was conducted according to the manufacturer' s instructions [20]. The h.all.v6.2.symbols.gmt was downloaded from the Molecular Signatures Database (http://www.broad.mit.edu/gsea/msigdb/). *P* < 0.05 and false discovery rate (FDR) of <0.05 were set as the enriched terms. According to the median value of KRT23, 308 OC patients in The Cancer Genome Atlas (TCGA) datasets were divided into a high-expression KRT23 group and a low-expression group. In addition, relative research on the relationship between KRT23 expression and molecules involved in the TGF-*β*/Smad signaling pathway was conducted on the Gene Expression Profiling Interactive Analysis (http://gepia.cancer-pku.cn/) platform.

### 2.2. Ovarian Tissue Samples

Patients diagnosed with OC underwent surgery at the Beijing Obstetrics and Gynecology Hospital. Fresh ovarian tumor tissues (*n* = 10) and adjacent normal tissues (*n* = 10) were obtained for subsequent analysis. The histology of the OC samples included serous (5), endometrioid (3), and clear cell (2) carcinoma. None of the patients received any chemotherapy or radiation treatment prior to surgery.

### 2.3. Immunohistochemistry

All OC tissues were immersed in 4% paraformaldehyde for 4 h and then embedded in paraffin. After that, the samples were cut into sections, incubated with primary antibodies of KRT23 (1 : 500 dilution) overnight at 4°C, and then with secondary antibodies for 1 h at 37°C. After washing, the slices were stained with 3,3-diaminobenzidin (DAB) and kept at room temperature without light for 10 min. The staining reaction was stopped with distilled water, followed by hematoxylin counterstaining. Ethanol and xylene were used for dehydration and transparency, respectively. Finally, the slices were sealed with neutral gum. Similar steps as described above were conducted for the negative control group, but KRT23 antibody was replaced with phosphate-buffered saline (PBS).

### 2.4. Cell Lines

The four human OC cell lines (HEY, SKOV3, OVCAR3, and CAOV3) were purchased from the National Infrastructure of Cell Line Resource (Beijing, China). The Human Ovarian Surface Epithelial Cells (HOSEpiC) were generously gifted by Dr. Liao. All the cell lines were cultured in Roswell Park Memorial Institute- (RPMI-) 1640 medium (Gibco, Gaithersburg, MD, USA), supplemented with 10% fetal bovine serum (FBS; HyClone Laboratories Inc., Logan, UT, USA) and 1% penicillin/streptomycin (Gibco, Gaithersburg, MD, USA), and then cultured in an incubator with a humidified atmosphere of 5% CO_2_ at 37°C.

### 2.5. RNA Extraction and Reverse Transcription Quantitative PCR (RT-qPCR)

Total cellular RNA was isolated using Trizol reagent (Invitrogen, Carlsbad, CA) according to the manufacturer's instructions. The concentration and purity of RNA were determined using a NanoDrop 1000 Spectrophotometer. The first strand of cDNA was synthesized using the ReverTra Ace qPCR RT kit (Toyobo, Shanghai, China) according to the manufacturer' s protocol. Real-time PCR analyses were performed using SYBR Premix EX Taq™ (Takara, Dalian, China) using an ABI 7500 Real-Time PCR system (Applied Biosystems, Foster City, USA). GAPDH expression was used to normalize the expression levels of KRT23 in each sample using the 2-*ΔΔ*Ct method. The primers for KRT23 and GAPDH were as follows:

KRT23 forward primer: 5′-CCATGCAGAATCTCAACGAC-3′ and reverse primer: 5′-GGTGTGTGATGTTTTCCTCA-3′;

GAPDH forward primer: 5′-TCAACGACCACTTTGTCAAGCTCA-3′ and reverse primer: 5′-GCTGGTGGTCCAGGGGTCTTACT-3′.

### 2.6. Western Blotting

HOSEpiC cells and OC cells were collected and lysed using lysis buffer containing radioimmunoprecipitation assay (RIPA) buffer (Thermo Fisher Scientific, Waltham, MA, USA), phenylmethanesulfonyl fluoride (PMSF), and protease inhibitor cocktail (Roche Diagnostics, Basel, Switzerland). The protein concentration was quantified using a bicinchoninic acid assay kit (Thermo Fisher Scientific, Waltham, MA, USA). Next, 10% sodium dodecyl sulfate/polyacrylamide gels (Gene Molecular Biotech Inc., Shanghai, China) were prepared to separate the protein samples. 30 micrograms of protein was loaded onto the gels and subsequently transferred onto polyvinylidene difluoride membranes (Gene Molecular Biotech, Inc., Shanghai, China). The membranes were incubated overnight at 4°C with primary antibodies (Cell Signaling Technology, Inc., Danvers, MA, USA) against KRT23 (dilution, 1 : 500), E-cadherin (dilution, 1 : 500), N-cadherin (dilution, 1 : 500), vimentin (dilution, 1 : 1000), Snail (dilution, 1 : 1000), Twist (dilution, 1 : 500), Smad2/3 (dilution, 1 : 1000), phosphorylated-Smad2/3 (dilution, 1 : 500), and GAPDH (dilution, 1 : 1000). TBST was used to wash the membranes, followed by incubation of the membranes with HRP-labeled secondary antibodies (dilution, 1 : 7500; Cell Signaling Technology, Inc., Danvers, MA, USA). The bands were detected using an enhanced chemiluminescence system (Roche Diagnostics, Basel, Switzerland). All experiments were repeated at least three times.

### 2.7. Small Interfering RNA Transfection

Three siRNAs were designed by JTSBIO Co., Ltd. (Wuhan, China) for KRT23 downregulation. The sequences of KRT23-target-specifc-siRNA (si-KRT23) were as follows: siRNA1: 5′-GCAGACAAGGTGACATCCA-3′; siRNA2: 5′-GGAGCGGCAGAACAATGAA-3′; and siRNA3: 5′-GGAGACCATCAACGGAAGA-3′. Nonsense RNAi was used as a control, and the sequence of control was 5′-UUCUCCGAACGUGUCAGGUTTUCCAGGTCUAGTT-3′. The cells were cultured at 2 × 10^5^ cells/mL in 6-well plates. The si-KRT23 and si-control were then transfected into cells using Lipofectamine™ RNAmax (Invitrogen, Carlsbad, CA, USA) according to the manufacturer's instructions. Transfection efficiency was verified by qRT-PCR and Western blotting after 48 h of transfection.

### 2.8. Wound Healing Assay

The cells obtained were seeded on 6-well plates and transfected with si-KRT23 or si-control. After that, a 100 *μ*L pipette tip was used to create a scratch wound on the cells. The medium was then replaced with serum-free medium, and cell migration was monitored every 24 h. Statistical analysis of cell migration was conducted using the ImageJ software.

### 2.9. Cell Migration Assay

The transwell (Corning Inc., NY, USA) migration assay was performed as reported previously [21]. Transwell chambers (Corning, Inc., NY, USA) were used for cell migration assays. The cells at a density of 2 × 10^4^ cells were seeded in the top chamber with 100 *μ*L serum-free medium per well, while the bottom chambers were filled with RPMI-1640 medium containing 10% FBS. After 24 h of migration, the invasive cells were fixed and counted. For cell invasion assays, transwells were coated with 50 *μ*L Matrigel (1 : 4 dilution with 0.2% BSA) (Sigma-Aldrich, St. Louis, MO, USA). Cells at a density of 1.2 × 10^5^ were seeded in the top layer of the transwell chambers per well, and the others were performed the same as described for the transwell migration assay.

### 2.10. Immunofluorescence

Cells transfected with si-KRT23 or si-control were cultured in 96-well plates (2 × 10^3^ cells per well), fixed in methanol, and permeabilized with 0.1% Triton X-100 in PBS for 10 min at room temperature. The cells were then incubated in the blocking buffer overnight at 4°C with the following antibodies (Cell Signaling Technology, Inc., Danvers, MA, USA) against Smad2 (dilution,1 : 200), Smad3 (dilution,1 : 200), phosphorylated Smad2 (dilution,1 : 200), and phosphorylated Smad3 (dilution,1 : 200). The cells were then rinsed three times with washing solution 3 times. FITC-conjugated secondary antibody (Solarbio, Beijing) was added to the wells and incubated for 1 h at room temperature. DAPI was used to stain the nuclear DNA. Imaging was performed using a Thermo Scientific™ CellInsight™ CX7 High Content Screening (HCS) Platform.

## 3. Results

### 3.1. KRT23 Overexpression in OC Tissues and Cell Lines

**Figure 1:**
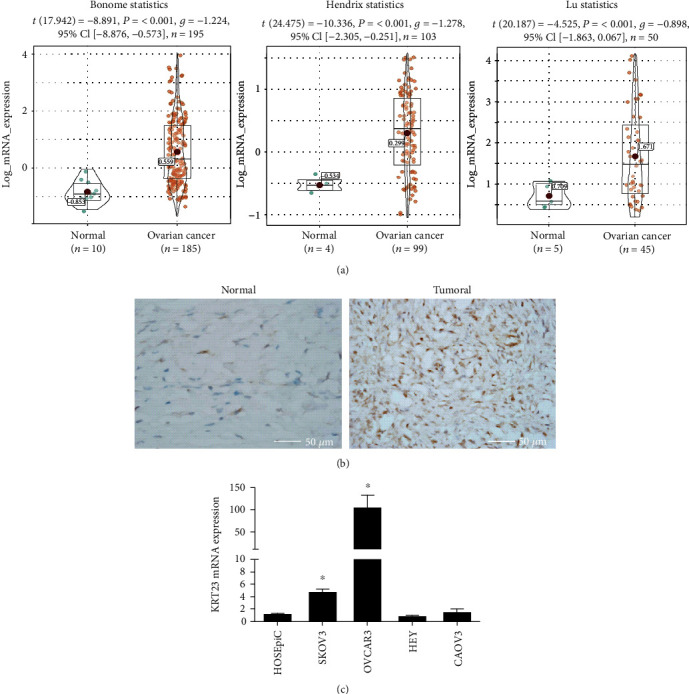
The expression of keratin 23 (KRT23) in ovarian cancer (OC) tissues and cell lines. (a) Gene expression datasets were analyzed in 329 OC tissues and 19 normal tissues that were obtained from the Oncomine database. KRT23 overexpression was found in OC patients. *P* values were calculated using Student's *t*-test. (b) Immunohistochemical staining of KRT23 in OC tissues (*n* = 10) and adjacent normal tissues (*n* = 10), and there were four pairs with positive KRT23 expression in ten paired samples. Scale bars, 50 *μ*m. (c) The levels of KRT23 in all OC cell lines were analyzed by reverse transcription quantitative polymerase chain reaction assay. The KRT23 level was higher in SKOV3 cells and OVCAR3 cells when compared with Human Ovarian Surface Epithelial Cells (HOSEpiC). Glyceraldehyde 3-phosphate dehydrogenase was used as an internal control. ^∗^*P* < 0.05.

To explore the expression of KRT23 in OC, the mRNA levels of KRT23 in 329 OC tissues and 19 normal tissues obtained from the Oncomine database were analyzed. Results showed that KRT23 mRNA levels in OC tissues were three times higher than that in normal tissues (Figure 1(a)). Concomitantly, the expression of KRT23 protein was tested in ten OC tissues and corresponding normal ovarian tissues by immunohistochemistry, and results showed that 70% (10/7) of OC tissues revealed significantly higher staining intensity of KRT23 than normal tissues (Figure 1(b)). Moreover, the expression of KRT23 was also tested in four human OC cell lines (HEY, SKOV3, OVCAR3, and Caov3) by RT-qPCR. As shown in Figure 1(c), overexpression of KRT23 was observed in two human OC cell lines (SKOV3 and OVCAR3) when compared with the human ovarian surface epithelial cell line (HOSEpiC). As the expression of KRT23 was higher in SKOV3 and OVCAR3 cell lines than in the other cell lines, the ovarian cell line SKOV3 was chosen for the following *in vitro* experiments. In summary, these data suggest that the expression of KRT23 was increased in OC tissues and cell lines.

### 3.2. Knockdown of KRT23 Inhibits OC Cell Migration and Invasion

**Figure 2:**
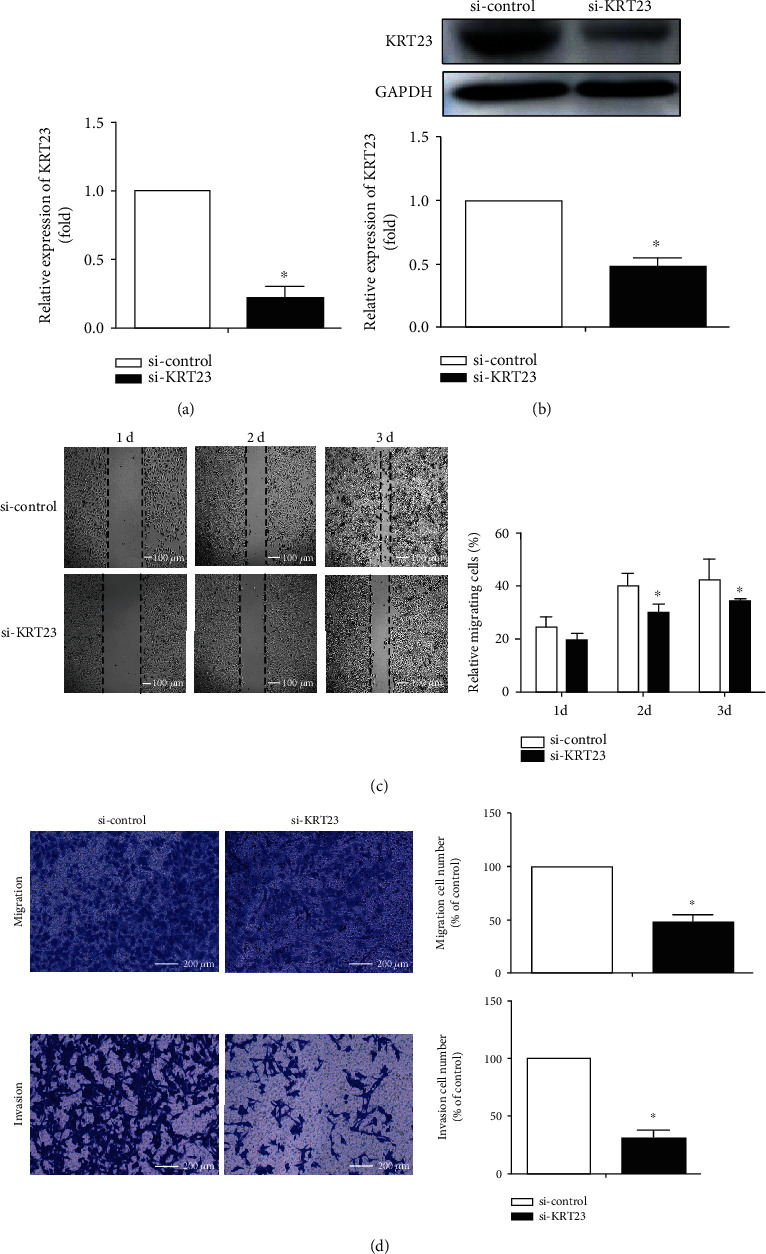
Silencing of keratin 23 (KRT23) expression decreased ovarian cancer (OC) cell migration and invasion. (a, b) The knockdown of KRT23 by small interfering RNA (siRNA) treatment was detected by reverse transcription quantitative polymerase chain reaction assay and Western blotting. KRT23 siRNA could efficiently decrease the expression of KRT23 in SKOV3 cells. ^∗^*P* < 0.05. (c, d) The migratory and invasive abilities of SKOV3 cells were evaluated by using wound healing and transwell assay, and knockdown of KRT23 significantly impaired the migratory and invasive abilities of SKOV3 cells. Scale bars, 200 *μ*m.^∗^*P* < 0.05.

To further explore the biological role of KRT23 in OC cells, the expression of KRT23 was knocked down in SKOV3 cells using a mixture of three different siRNAs (siRNA1, siRNA2, and siRNA3). The efficiency of transfection was verified by Western blotting and RT-qPCR, and the results showed that these siRNA constructs effectively knocked down both KRT23 RNA and protein levels by more than 80% (Figures 2(a) and 2(b)). The wound healing assay showed that KRT23 silencing impaired the migratory ability of SKOV3 cells, and therefore, the number of migrating cells in the KRT23 knockdown group was less than that in the control group by 26% and 19%, at 48 h and 72 h after transfection with si-KRT23, respectively (Figure 2(c)). Similarly, cell migration and invasion tests demonstrated that the number of cells that migrated and invaded through the 8 *μ*m microwells was significantly reduced by 56% and 75%, respectively, in the KRT23 knockdown group when compared with the control group (Figure 2(d)). These data indicate that KRT23 could promote OC migration and invasion *in vitro*.

### 3.3. KRT23 Promotes EMT in OC Cells

**Figure 3:**
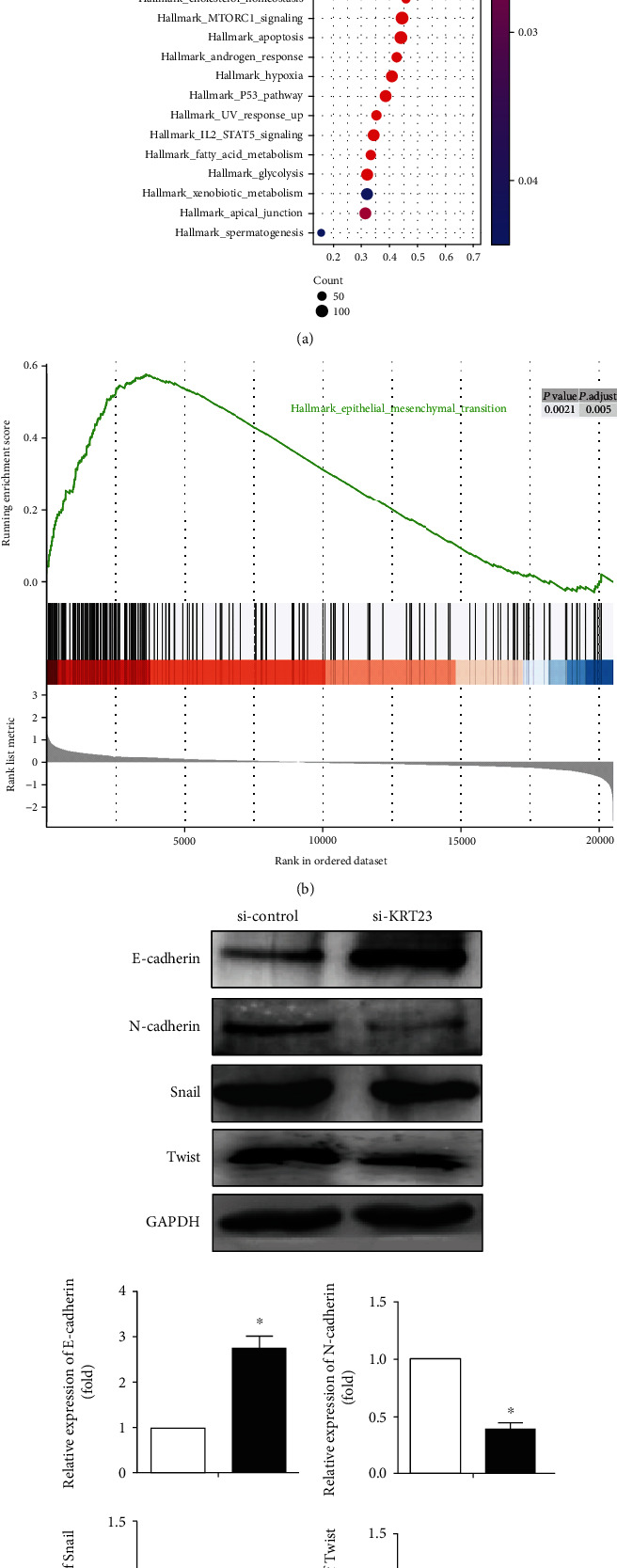
The effects of keratin 23 (KRT23) on epithelial-to-mesenchymal transition (EMT) progress. (a, b) The Gene Set Enrichment Analysis (GSEA) of EMT in 154 samples exhibited high KRT23 expression and in the remaining 154 samples exhibited low KRT23 expression, which indicated that the high KRT23 expression was related to EMT. (c) The expression of EMT markers was detected by Western blotting analysis. Knockdown of KRT23 increased the expression level of E-cadherin but reduced the expression levels of N-cadherin, Snail, and Twist when compared with the control group.

Gene Set Enrichment Analysis (GSEA) was used to explore the potential mechanism of KRT23 upregulation in OC [20]. A total of 308 OC tissues obtained from TCGA database were divided into 154 samples exhibiting high KRT23 expression and 154 samples exhibiting low KRT23 expression according to the median value of KRT23, and data were submitted to the GSEA. As shown in Figures 3(a) and 3(b), results indicated that KRT23 overexpression may be involved in EMT, which refers to the transfer of epithelial cells to mesenchymal cells morphologically, and occurs before the tumor cells acquire migratory and invasive abilities [22, 23]. Findings from our study revealed that the expression of KRT23 was increased in OC tissues and cell lines, and knockdown of KRT23 inhibited OC cell migration and invasion. Therefore, it is necessary to explore whether KRT23 silencing could affect the expression of several markers of EMT. As expected, the Western blot assay yielded results consistent with GSEA, as the expression of epithelial marker E-cadherin was increased in the cells transfected with si-KRT23, whereas the expression of mesenchymal markers vimentin and N-cadherin were decreased (Figure 3(c)). These results suggest that KRT23 influenced the process of EMT in OC cells.

### 3.4. KRT23 Promotes EMT via the TGF-*β*/Smad Signaling Pathway

**Figure 4:**
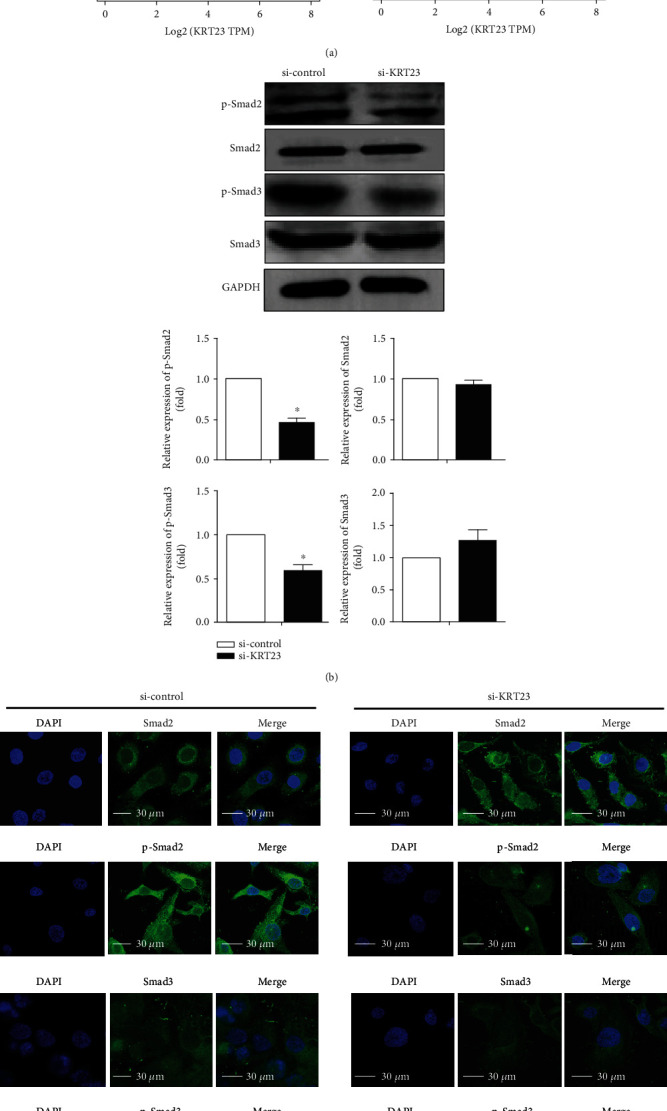
Keratin 23 (KRT23) expression affected TGF-*β*/Smad signaling pathways. (a) Scatter plots of KRT23 and genes involved in TGF-*β*/Smad signaling pathways. (b) Western blot was conducted to analyze the expression of TGF-*β*/Smad signaling pathway-related genes. KRT23 knockdown reduced the expression levels of p-Smad2/p-Smad3. (c) SKOV3 cells were transfected with control or KRT23 siRNA as indicated. Cells were then subjected to immunofluorescence using Smad2, p-Smad2, Smad3, and p-Smad3 antibodies and DAPI. Immunofluorescence staining showed that after silencing KRT23 expression, the expression of p-Smad2/p-Smad3 decreased significantly. Scale bars, 30 *μ*m.

Among the pathways that involve the EMT of tumor cells, the TGF-*β* signaling pathway plays an important role in inducing EMT, thus promoting tumor cell invasion and migration [24]. GEPIA database analysis revealed a positive correlation between KRT23 and Smad2/Smad3 involved in the TGF-*β*/Smad signaling pathway (Figure 4(a)). To further investigate the effect of KRT23 on TGF-*β*/Smad-mediated EMT, SKOV3 cells were pretreated with KRT23 siRNA, and the expression of TGF-*β*/Smad signaling pathway-related molecules was examined by Western blotting. As shown in Figure 4(b), KRT23 depletion decreased p-Smad2/p-Smad3 expression. Furthermore, immunofluorescence staining was conducted to verify these results in SKOV3 cells. Consistently, the downregulation of KRT23 attenuated the expression of p-Smad2/p-Smad3 (Figure 4(c)). Taken together, these results suggest that KRT23 promoted EMT by regulating the TGF-*β*/Smad signaling pathway.

## 4. Discussion

Cytokeratin genes are the most common and specifically expressed genes in epithelial cells, which influence cell proliferation, invasion, and migration in different types of cancers. KRT23 is a newly discovered member of the keratin family. In the present study, KRT23 upregulation in OC promoted cell migration and invasion via EMT by regulating the TGF-*β*/Smad signaling pathway.

Previous studies have presented evidence of aberrant expression of KRT23 in various tumor tissues, including pancreatic cancer, colorectal carcinoma, and hepatocellular carcinoma [13–16]. We have shown, for the first time, that OC tissues and cell lines have high KRT23 expression. We conducted *in vitro* cellular experiments to explore the role and function of KRT23 in OC. KRT23 was reported to be involved in cell migration and cell invasion in colorectal cancer [15]. According to our results, KRT23 knockdown impaired OC cell migration and invasion. By utilizing GSEA, we showed that the underlying mechanism of KRT23 knockdown in OC is via influencing the EMT, resulting in the decreased migratory ability of OC cells.

Various studies have shown that EMT is the central mechanism responsible for cell migration and invasion in various cancers, including OC. After EMT, the invasive ability of tumor cells was significantly enhanced. The role of EMT in the invasion and migration of OC has been widely verified. Blechschmidt et al. found that the expression of E-cadherin and Snail was negatively correlated in OC and was related to cell invasion and migration [25]. The expression of Snail was significantly increased in advanced primary tumors and lymph node metastases of OC patients, and it could participate in the invasion and migration of OC cells by activating matrix metalloproteinases [26]. Gao et al. have shown that nucleus accumbens-1 (NAC1) is upregulated in OC tissues. NAC1 overexpression promoted the expression of twist and inhibited the expression of E-cadherin, leading to the occurrence of EMT in OC and increased cell migratory and invasive abilities [27]. In our study, we found that knockdown of KRT23 increased the expression level of epithelial marker E-cadherin but reduced the expression levels of mesenchymal markers N-cadherin, Snail, and Twist. These results demonstrate that KRT23 is involved in the EMT process.

The transforming growth factor-beta (TGF-*β*) signaling pathway regulates various biological processes, including cell growth, cell differentiation, and extracellular matrix remodeling [28]. Upon ligand binding to TGF-*β* receptors, Smad2/3 is activated, which then actively phosphorylates Smad2/3 and Smad4, forming a complex and translocating into the nucleus. The complex in the nucleus binds to a site called Smad-binding element (SBE) within the gene promoter region and controls gene expression [29]. Abnormalities in the TGF-*β* signaling pathway are linked to a variety of human diseases, including cancer, inflammation, and tissue fibrosis [30, 31]. TGF-*β* inhibits or promotes tumor invasion and migration [32]. Experiments have shown that TGF-*β* is a major factor that promotes EMT in multiple tumors [33, 34]. Previous studies have provided convincing evidence that keratin participates through the EMT of tumors by regulating the TGF-*β* signaling pathway. Fang et al. reported that KRT8 overexpression was related to EMT in promoting tumor progression and migration of gastric cancer by activating integrin b1-FAK and TGF-*β*/Smad signaling pathways [35]. Promoter binding analysis revealed that Smad4 was associated with KRT23 expression in migratory colon cancer cells, and KRT23 exhibited Smad4-dependent upregulation, thus influencing cell signal transduction and eventually promoting colon cancer cell migration [19]. However, the precise molecular mechanism by which KRT23 regulates EMT in OC cells remains unclear. In this study, bioinformatic analysis suggested that KRT23 may accelerate EMT by regulating the TGF-*β* signaling pathway. Furthermore, we also found that the expression of p-Smad2 and p-Smad3 was decreased in KRT23-siRNA-treated SKOV3 cells when compared with controls. These results demonstrate that KRT23 participates in the TGF-*β* signaling pathway-mediated EMT. However, TGF-*β* signaling pathway is a complex signal network mediated through its transmembrane receptor and subsequent activation of cytoplasmic Smad proteins, the precise mechanisms by which KRT23 regulates the TGF-*β* signaling pathway remain unclear. Whether KRT23 could directly interact with molecules in the TGF-*β* signaling pathway (such as Smad2/3) or modify the expressions of TGF-*β* receptors needs to be further verified by experiments. The next question that could be addressed is the potential role of KRT23 in OC metastasis in vivo.

## 5. Conclusions

These data demonstrated that the expression of KRT23 was increased in OC tissues and OC cell lines. KRT23 overexpression promoted cell migration and EMT progression by regulating the TGF-*β* signaling pathway. Further investigation is warranted to verify the role of KRT23 *in vivo*.

## Data Availability

The datasets used and/or analyzed during the current study are available from the corresponding author upon reasonable request.
